# The association between XPD rs13181 and rs1799793 polymorphism and oral cancer risk: evidence from a meta-analysis

**DOI:** 10.1186/s12885-024-12503-3

**Published:** 2024-06-15

**Authors:** Wenli Zeng, Wanting Xu, Wu Long

**Affiliations:** 1 Department of Stomatology, The People’s Hospital of Yichun City, Yichun, Jiangxi 336028 China; 2 Department of Stomatology, The Second People’s Hospital of Yichun City, Yichun, Jiangxi 336028 China

**Keywords:** XPD, rs13181, rs1799793, SNP, Oral cancer, Meta-analysis

## Abstract

**Objective:**

Single nucleotide polymorphisms (SNPs) are common in genes and can lead to dysregulation of gene expression in tissues, which can affect carcinogenesis. Many studies reporting the association between xeroderma pigmentosum group D (XPD) polymorphisms of rs13181 and rs1799793 with oral cancer risk, but with conflicting and inconclusive results.

**Methods:**

We performed a comprehensive and systematic search through the PubMed, Elsevier, Web of science, and Embase databases, twelve studies were included in the meta-analysis to determine whether XPD rs13181 and rs1799793 polymorphism contributed to the risk of oral cancer.

**Results:**

The pooled date indicated a significant association between the rs13181 polymorphism and oral cancer risk for the allele comparison model (odds ratio, OR = 1.60, 95% confidence intervals, CI = 1.09–2.35, *P* = 0.02), the dominant model (OR = 1.74, 95% CI = 1.08–2.82, *P* = 0.02), and the heterozygote model (OR = 1.59, 95% CI = 1.02–2.49, *P* = 0.04). For the XPD rs1799793 polymorphism, it is not associated with the incidence of oral cancer under any model. Subgroup analyses based on ethnicity indicated that the rs13181 polymorphism increased the risk of oral cancer among Asians according to the allele comparison model (OR = 1.97, 95% CI = 1.10–3.51, *P* = 0.02), the dominant model (OR = 2.35, 95% CI = 1.25–4.44, *P* = 0.008), the heterozygote model (OR = 2.05, 95% CI = 1.15–3.66, *P* = 0.01), and the homozygous model (OR = 2.47, 95% CI = 1.06–5.76, *P* = 0.04).

**Conclusion:**

Our meta-analysis suggests a positive correlation between XPD rs13181polymorphism and the development of oral cancer among Asians, but a negative correlation among Caucasians populations.

## Introduction

In the last few decades, there has been an increasing incidence of oral cancer worldwide, with an estimated 377,713 new cases and 177,757 deaths recorded in 2020 [1]. The development of oral cancer is influenced by many factors such as diet and nutrition, familial and genetic predisposition, tobacco, alcohol, use of mouthwash, radiation, oral thrush, viruses, syphilis, immunosuppression, dental factors, occupational risks, and mate [2]. The genetic susceptibility factors of oral cancer are determined by family inheritance and ethnic characteristics, and many studies have shown that genetic polymorphism plays an important role in the occurrence and development of oral cancer [3–6]. Single nucleotide polymorphisms (SNPs) are common in genes and can lead to dysregulation of gene expression in tissues, which can affect carcinogenesis [5].

Xeroderma pigmentosum group D (XPD), also known as excision repair cross-complementation rodent repair deficiency group 2 (ERCC2), is located on chromosome 19q13.3, comprises of 23 exons and encodes 760 amino acids [7]. The XPD gene functions in the nucleotide excision repair (NER) pathway, alterations in this gene can cause defective DNA repair efficiency, ultimately leading to genomic instability and carcinogenesis [8]. Two SNPs in XPD have been shown to be involved in susceptibility to oral cancer: codon 312 (G > A substitution at position 23,951, exon 10, Asp > Asn, rs1799793) and codon 751 (A > C substitution at nucleotide position 35,931, exon 23, Lys > Gln, rs13181) [9]. The Lys751Gln variant affects an ATP-binding site of XPD and impairs its helicase activity, which is important for NER, but does not affect its transcriptional activity [7]. The function of Asp312Asn remains to be elucidated. Many studies have reported the association between polymorphisms of rs13181 and rs1799793 with oral cancer risk, but with conflicting and inconclusive results [7, 8, 10–19]. Thus, we performed this comprehensive meta-analysis to better illustrate the relationship between XPD polymorphism and oral cancer risk.

## Materials and methods

### Publication search strategy

A comprehensive and systematic search through the PubMed, Elsevier, Web of science, and Embase databases was performed using the following terms: “XPD” or “ERCC2”, “polymorphism” or “gene mutation” or “gene variation”, “oral cancer”, “oral neoplasms”, “mouth neoplasms” or “oral carcinoma”. The last search was updated on May 19th, 2024. All relevant publications were reviewed and the reference lists of articles were also searched for potentially relevant publications. There was no language or sample size limitations in the included studies. The study was registered in the International prospective register of systematic review (ID: 550,899).

### Inclusion and exclusion criteria

Studies included should meet the following criteria: (1) cohort study or case–control study; (2) evaluation of XPD polymorphism and oral cancer risk; and (3) sufficient data to examine odds ratio (OR) with 95% confidence interval (95% CI). Major criteria for exclusion were as follows: (1) oral potentially malignant disorders not for oral cancer research; (2) studies not focused on XPD polymorphism; (3) studies with insufficient data for analysis or duplicated data; (4) reviews or meta-analyses; (5) studies where the distribution of genotypes among controls is not in Hardy–Weinberg equilibrium.

### Data extraction

The eligible data in the studies were extracted by two investigators, and consensus was reached through discussion when divergences appeared. The following information was extracted: first author’s name, year of publication, ethnicity, numbers of cases and controls with the AA, AC, and CC genotypes for rs13181, the GG, GA, and AA genotypes for rs1799793, and the sample size of cases and controls. The ethnic populations were classified as either Asian or Caucasian.

### Statistical analysis

We evaluated the risk using the allele comparison model (A vs. G for rs1799793; C vs. A for rs13181), the dominant model (GA + AA vs. GG for rs1799793; AC + CC vs. AA for rs13181), the recessive model (AA vs. GA + GG for rs1799793; CC vs. AC + AA for rsrs13181), the heterozygote model (GA vs. GG for rs1799793; AC vs. AA for rs13181), and the homozygote model (AA vs. GG for rs1799793; CC vs. AA for rs13181). Odds ratio (OR) with 95% confidence intervals (CI) were analyzed to measure the strength of association between the XPD rs13181 and rs1799793 polymorphism with oral cancer risk. The weighted mean difference with 95% CI was calculated. The statistical significance of the pooled OR was assessed using a Z test with a two-tailed *P* value of < 0.05 considered to be statistically significant. Stratified analysis by ethnicity was also carried out. Statistical heterogeneity, funnel plots, sensitivity analysis, and publication bias were analyzed as previously described [20]. All statistical tests for this meta-analysis were performed with Review Manager (RevMan) [Computer program]. Version5.4, The Cochrane Collaboration, 2020. and Stata Statistical Software 12 (StataCorp., T.X., USA).

## Results

### Characteristics of included studies

**Fig. 1:**
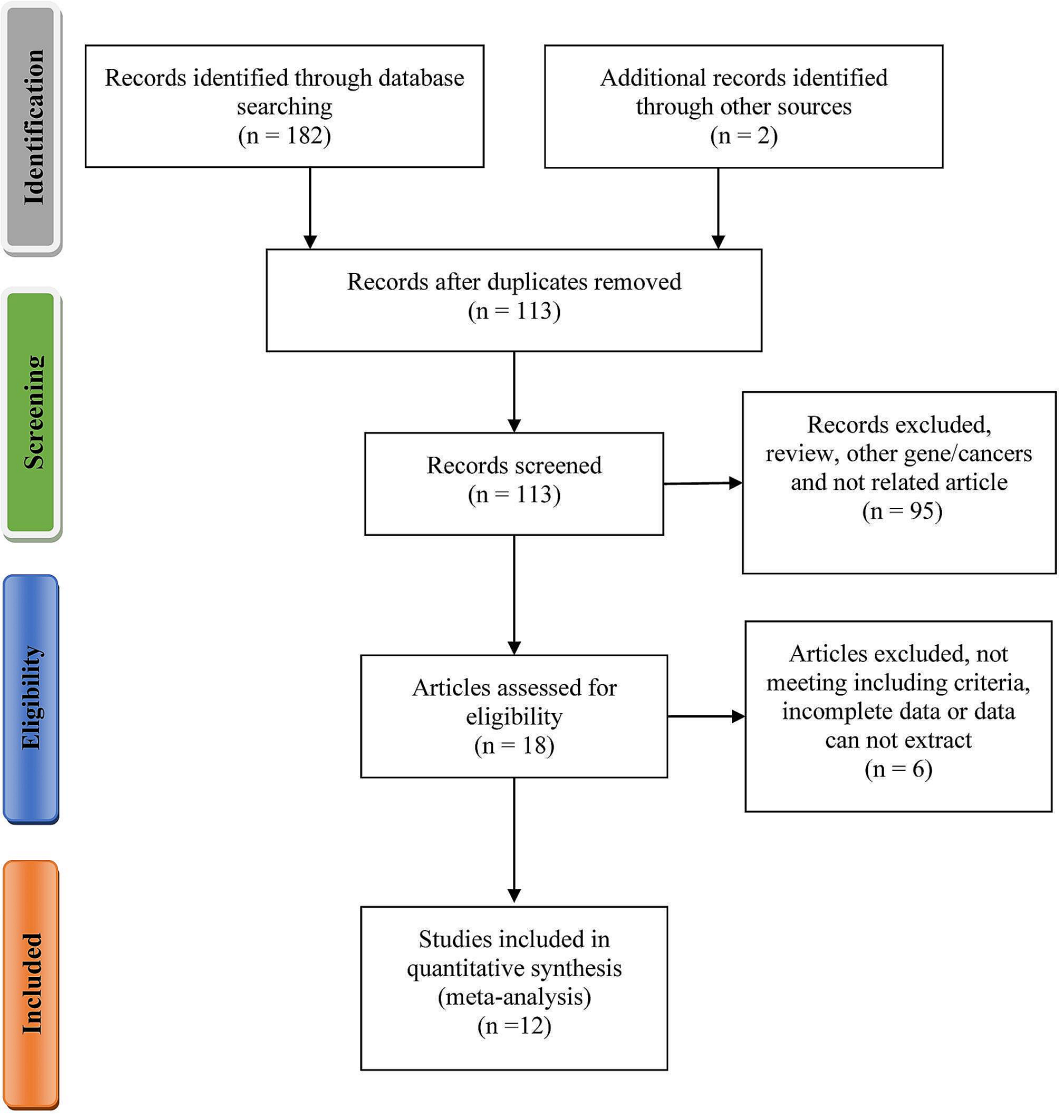
Flow diagram of the details of the study

A total of one hundred and eighty-two records were identified based on the search strategy, and two additional records were added after screening reference lists. One hundred and thirteen records were screened, and twelve studies were included in this meta-analysis [7, 8, 10–19] (Fig. 1). A total of 1725 patients with oral cancer and 1833 controls were tested for the XPD rs13181 polymorphism, of which 476 patients with oral tumors and 491 controls were Caucasian (Table 1). Three studies including 659 oral cancer patients and 741 controls, focused on XPD rs1799793 polymorphism [7, 15, 19] (Table 2).

**Table 1:** Main characters of studies included in the meta-analysis (XPD rs13181)

First author	Year	Ethnicity	Sample size		Cases			Controls		
			Case	Control	Lys751Lys	Lys751Gln	Gln751Gln	Lys751Lys	Lys751Gln	Gln751Gln
Buch	2005	Caucasian	273	269	104	125	44	153	84	32
Kietthubthew	2006	Asian	105	164	83	21	1	126	36	2
Ramachandran	2006	Asian	110	110	49	46	15	71	31	8
Bau	2007	Asian	154	105	134	18	2	89	15	1
Majumder	2007	Asian	309	388	158	125	26	190	158	40
dos	2016	Caucasian	54	40	23	24	7	10	28	2
Avci	2018	Caucasian	111	148	32	68	11	54	66	28
Nigam	2019	Asian	296	300	98	127	71	70	146	84
Tejasvi	2020	Asian	150	150	106	38	6	135	14	1
Galíndez	2021	Caucasian	38	34	12	22	4	14	17	3
Shrivastava	2022	Asian	50	50	12	32	6	47	2	1
Tata	2022	Asian	75	75	15	20	40	48	18	9

**Table 2:** Main characters of studies included in the meta-analysis (XPD rs1799793)

First author	Year	Ethnicity	Sample size		Cases			Controls		
			Case	Control	Asp312Asp	Asp312Asn	Asn312Asn	Asp312Asp	Asp312Asn	Asn312Asn
Buch	2005	Caucasian	204	204	88	93	23	110	77	17
Majumder	2007	Asian	305	387	152	119	34	205	146	36
Tejasvi	2020	Asian	150	150	86	60	4	126	22	2

### Quantitative synthesis

**Fig. 2:**
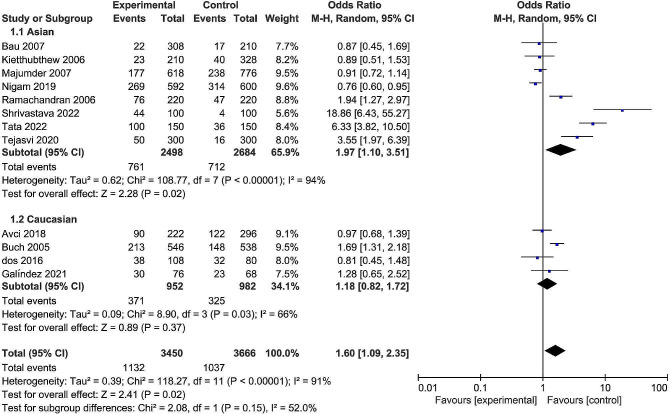
Forest plot for the meta-analysis of the association between XPD rs13181 polymorphism and oral cancer risk (under allele comparison model)

**Fig. 3:**
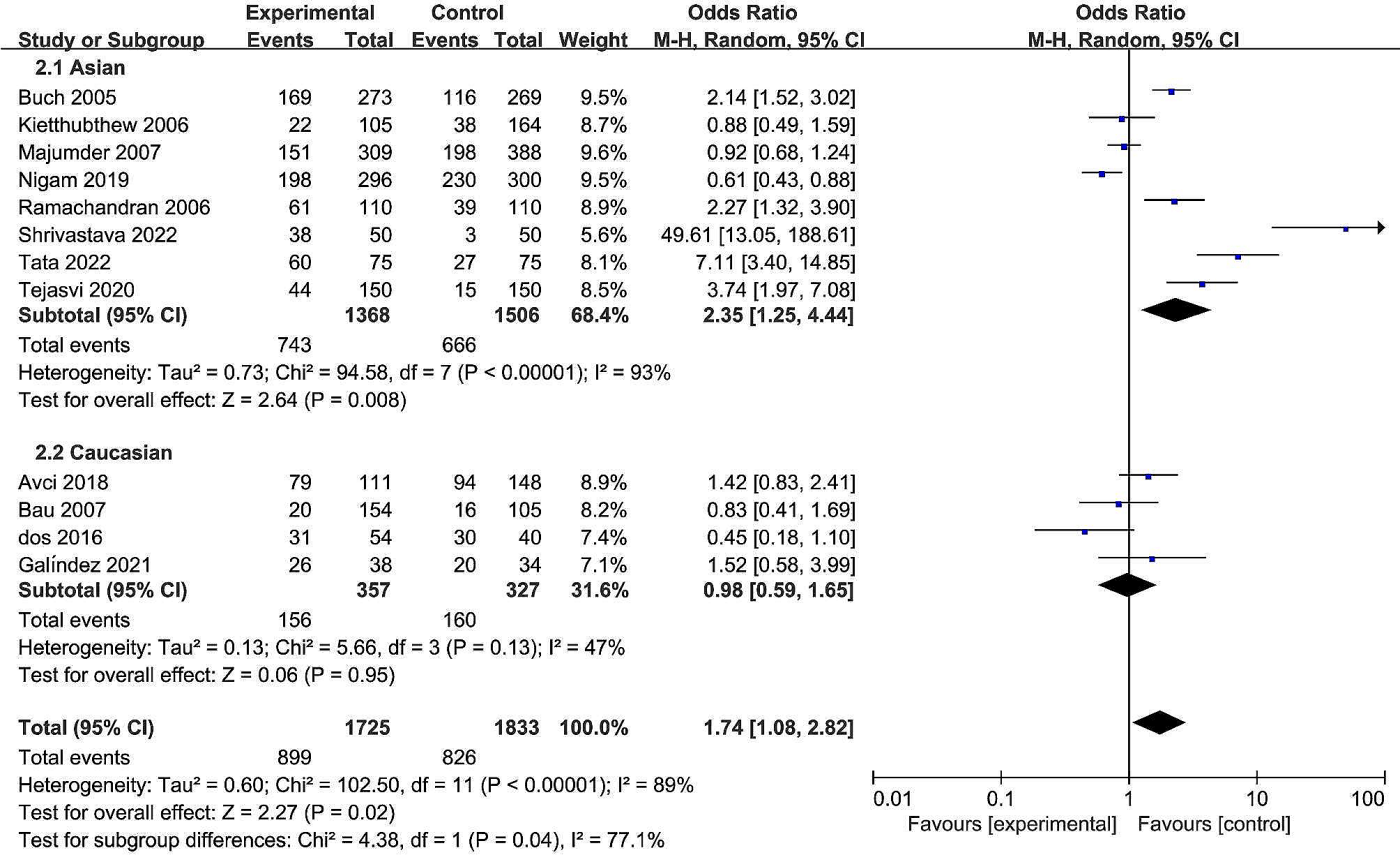
Forest plot for the meta-analysis of the association between XPD rs13181 polymorphism and oral cancer risk (under dominant comparison model)

**Fig. 4:**
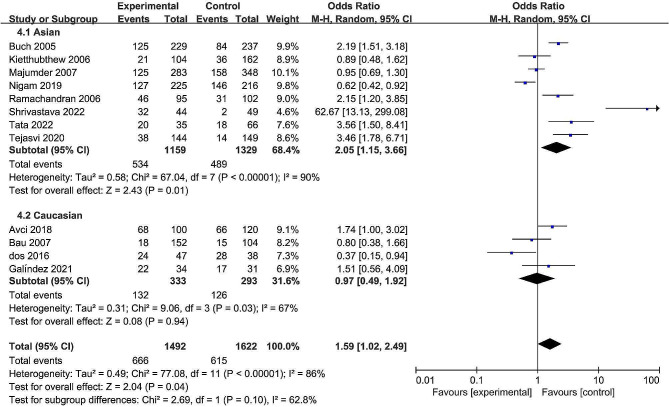
Forest plot for the meta-analysis of the association between XPD rs13181 polymorphism and oral cancer risk (under heterozygote comparison model)

**Fig. 5:**
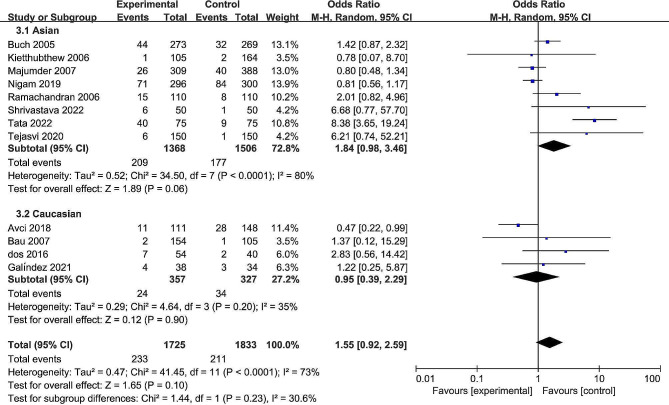
Forest plot for the meta-analysis of the association between XPD rs13181 polymorphism and oral cancer risk (under recessive comparison model)

**Fig. 6:**
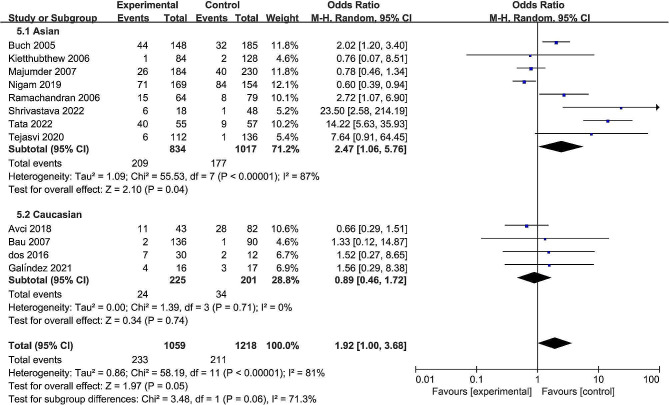
Forest plot for the meta-analysis of the association between XPD rs13181 polymorphism and oral cancer risk (under homozygous comparison model)

**Fig. 7:**
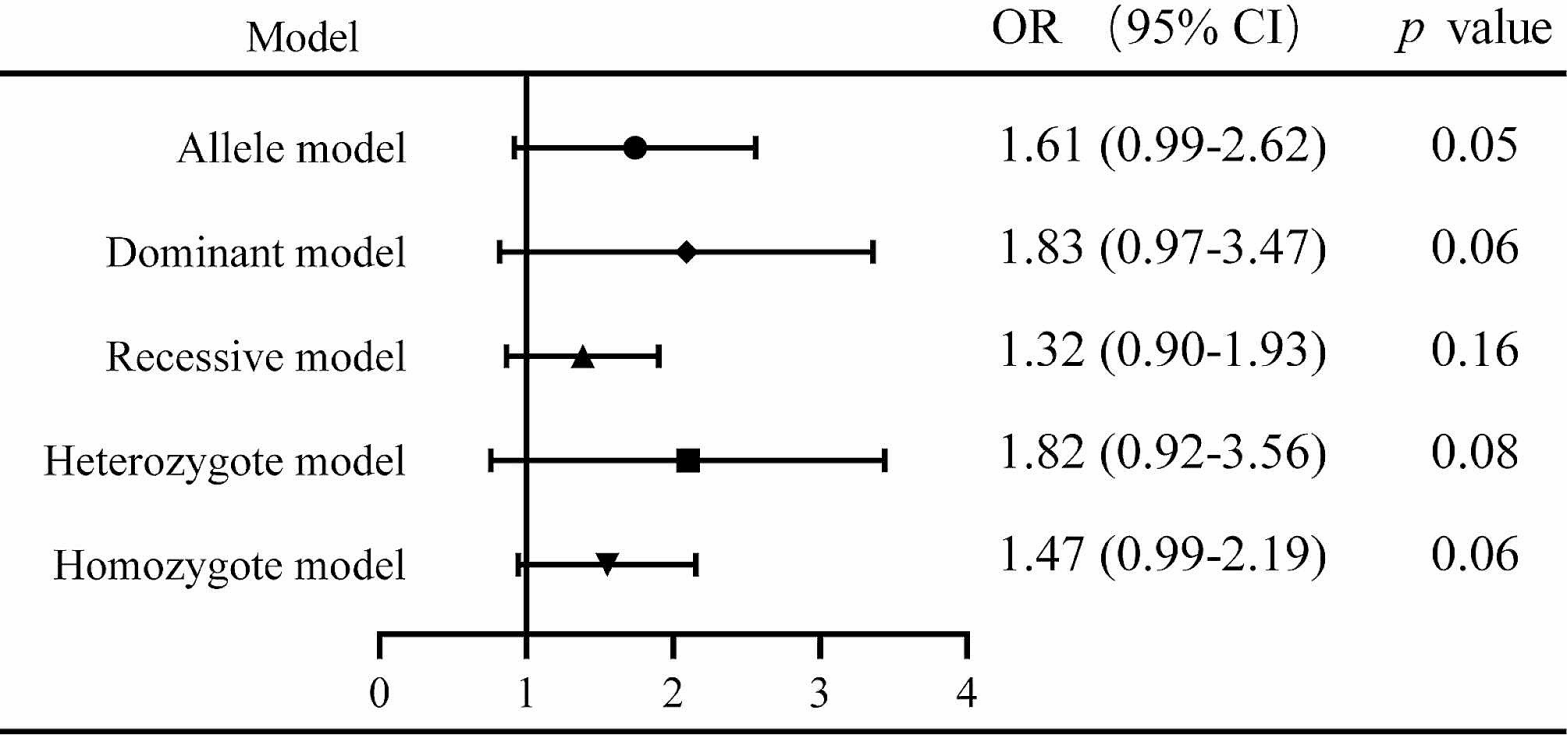
Results for the meta-analysis of the association between XPD rs1799793 polymorphism and oral cancer risk

The pooled date indicated a significant association between the rs13181 polymorphism and oral cancer risk in the allele comparison model (OR = 1.60, 95% CI = 1.09–2.35, *P* = 0.02, Fig. 2), dominant model (OR = 1.74, 95% CI = 1.08–2.82, *P* = 0.02, Fig. 3), and heterozygote model (OR = 1.59, 95% CI = 1.02–2.49, *P* = 0.04, Fig. 4), but not in the recessive model (OR = 1.55, 95% CI = 0.92–2.59, *P* = 0.10, Fig. 5) and homozygous model (OR = 1.92, 95% CI = 1.00-3.68, *P* = 0.05, Fig. 6). Subgroup analyses were carried out according to the ethnicity, revealing that the rs13181 polymorphism increased the risk of oral cancer among Asians in the allele comparison model (OR = 1.97, 95% CI = 1.10–3.51, *P* = 0.02, Fig. 2), dominant model (OR = 2.35, 95% CI = 1.25–4.44, *P* = 0.008, Fig. 3), heterozygote model (OR = 2.05, 95% CI = 1.15–3.66, *P* = 0.01, Fig. 4), and homozygous model (OR = 2.47, 95% CI = 1.06–5.76, *P* = 0.04, Fig. 6). No association was found between the rs13181 polymorphism and oral cancer risk among the Caucasian population. For the XPD rs1799793 polymorphism under any model, there was no association with the incidence of oral cancer (Fig. 7).

### Sensitivity analysis

**Fig. 8:**
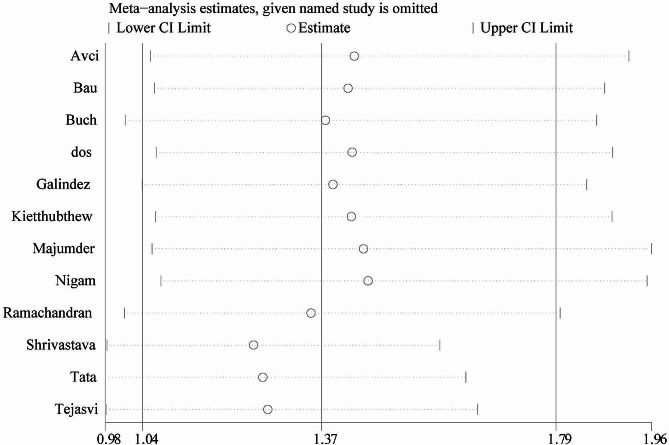
One-way sensitivity analysis of the pooled ORs and 95% CI, omitting each dataset in the meta-analysis. (under allele comparison model)

In the sensitivity analysis, each individual study included in the meta-analysis was sequentially removed to observe its influence on the pooled ORs. We found that two studies significantly affected the pooled OR value [8, 10], suggesting relative instability in the results of this meta-analysis (Fig. 8 for allele model).

### Publication bias

**Fig. 9:**
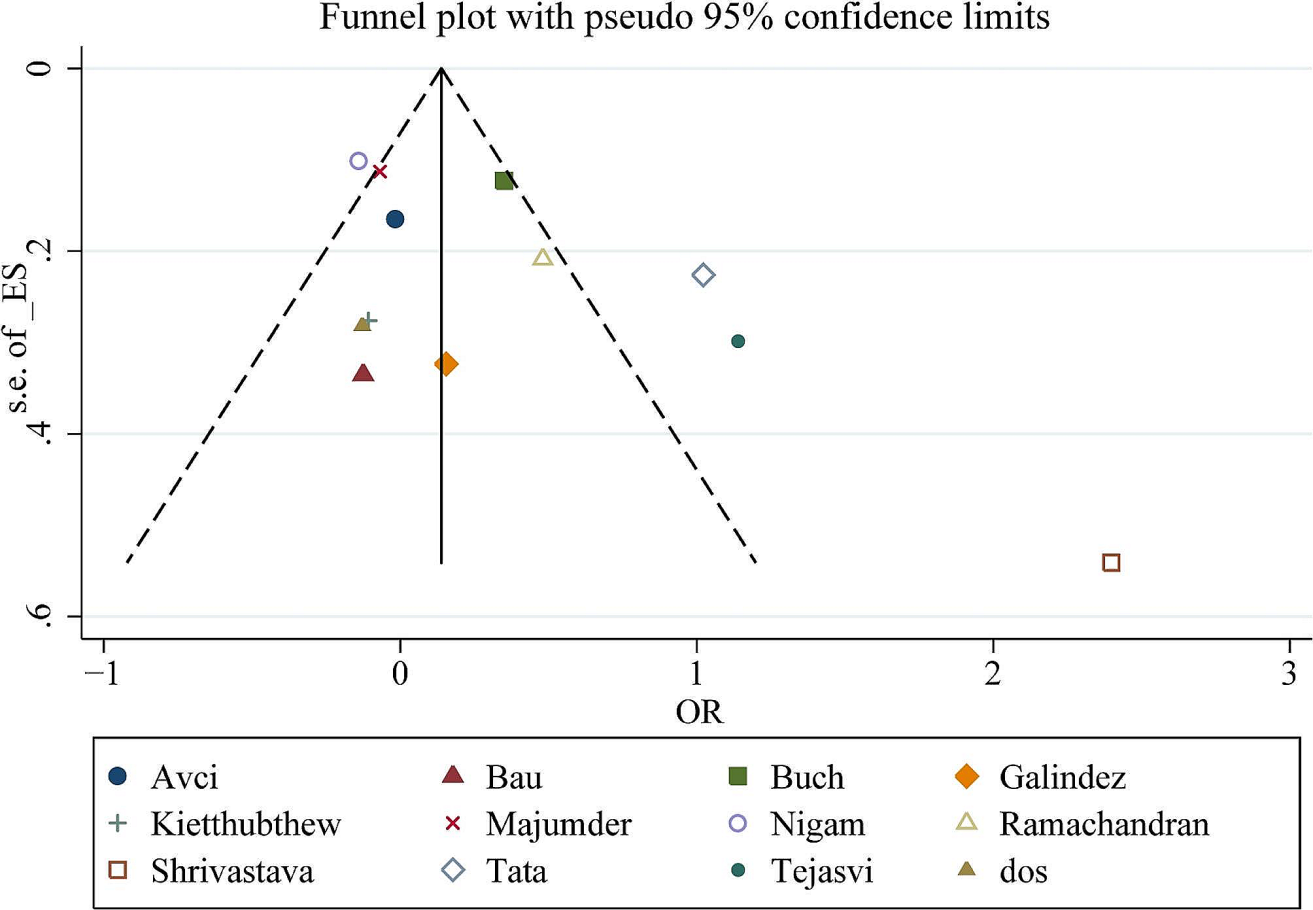
Funnel plot for the meta-analysis of the association between XPD rs13181 polymorphism and oral cancer risk. (under allele comparison model)

The symmetric shape of funnel plots suggested low publication bias (Fig. 9 for allele model). Begger’s funnel plot and Egger’s test were also performed. The statistical results showed no significant publication bias (*P* = 0.064 for allele model). Egger’s test was also conducted to assess the publication bias (*P* = 0.066 for allele model).

## Discussion

Defects in DNA repair mechanisms can lead to genomic instability and increased cell proliferation, which are significant factors in caner development [21]. XPD is a crucial component of the mammalian transcription factor II H (TFIIH), involved in eukaryotic transcription initiation and DNA nucleotide excision repair processes. Research suggests that XPD polymorphisms directly impact TFIIH complex activity, leading to abnormal DNA repair and transcription defects [22]. Studies have linked XPD gene polymorphisms to various malignancies, including liver cancer, gastrointestinal tumors, and oral cancer [10, 23, 24].

To our knowledge, this meta-analysis is the first comprehensive examination of XPD rs13181 and rs1799793 polymorphisms in relation to oral cancer risk. Our findings indicate an association between XPD rs13181, but not rs1799793, polymorphism and oral cancer risk, particularly among Asian populations. The genetic polymorphism may increase the risk of oral cancer by 1.59 to1.74 times. Interestingly, our results were not consistent with those of previous meta-analyses. While pooled odds ratio from a study comprising 1093 cases and 2637 controls found no association between XPD Lys751Gln polymorphism and oral cancer risk across all genetic models [21], another meta-analysis including 1202 cases and 1145 controls indicated no significant associations between XPD rs1799793 and rs13181 polymorphisms and overall oral cancer risk. However, the rs13181 polymorphism might be associated with oral leukoplakia risk [9]. Our meta-analysis, incorporating 12 studies with a total of 1725 cases and 1833 controls, offers more extensive data than previous analyses. However, limitations persist. Limited data from Caucasian populations hindered subgroup analysis, as only one relevant study was found in a meta-analysis [9], and incomplete data were noted in another [21, 25].

Early detection and treatment of oral lesions can reduce the progression to oral cancer [12]. Our study suggests that screening for XPD rs13181 polymorphism in Asian populations may aid in early detection of oral cancer, facilitating timely intervention. Moreover, XPD rs13181 and rs1799793 polymorphisms may predict clinical outcomes in oral cancer patients undergoing postoperative radiotherapy [26]. Additionally, these polymorphisms could influence the clinical sensitivity of platinum-based chemotherapy [7], though limited data prevented meta-analysis.

While our meta-analysis provides valuable insights, it has limitations. Sensitivity analysis revealed relative instability, possibly due to two influential studies [8, 10], necessitating further more case-control studies. Factors such as gender, family history, environmental influences, and lifestyle, including tobacco and alcohol consumption, were not evaluated in our analysis, despite their known association with oral cancer [12]. Additionally, the sample size was relatively small, though larger than in previous meta-analyses [9, 21]. Although no publication bias was observed, positive results may be more likely to be published, potentially affecting the meta-analysis [20]. Despite these limitations, our study offers a comprehensive exploration of the relationship between XPD polymorphisms and oral cancer susceptibility.

## Conclusion

In summary, our meta-analysis suggested that the XPD rs13181 polymorphism, but not rs1799793, is associated with oral cancer risk. Future studies with well-designed and larger population studies are needed to confirm these findings.

## Data Availability

The datasets used during the current study are available from the corresponding author on reasonable request.

## Abbreviations

SNPs: Single nucleotide polymorphisms
XPD: Xeroderma pigmentosum group D
ERCC2: Excision repair cross-complementation rodent repair deficiency group 2
OR: Odd ratio
NER: Nucleotide excision repair
